# SARS-CoV-2 Sero-Surveillance in Greece: Evolution over Time and Epidemiological Attributes during the Pre-Vaccination Pandemic Era

**DOI:** 10.3390/diagnostics12020295

**Published:** 2022-01-25

**Authors:** Michalis Koureas, Zacharoula Bogogiannidou, Alexandros Vontas, Maria A. Kyritsi, Varvara A. Mouchtouri, Katerina Dadouli, Lemonia Anagnostopoulos, Paraskevi Mina, Alexia Matziri, Maria Ntouska, Maria Tsigaridaki, Vasiliki Gkiata, Konstantinos K. Tsilidis, Evangelia E. Ntzani, Panagiotis Prezerakos, Sotirios Tsiodras, Matthaios Speletas, Christos Hadjichristodoulou

**Affiliations:** 1 Laboratory of Hygiene and Epidemiology, Faculty of Medicine, University of Thessaly, 41222 Larissa, Greece; mkoureas@med.uth.gr (M.K.); xara.16.01@gmail.com (Z.B.); avontas@uth.gr (A.V.); mkiritsi@med.uth.gr (M.A.K.); mouchtourib@med.uth.gr (V.A.M.); katerina1dad@gmail.com (K.D.); lanagnost@uth.gr (L.A.); pmina@med.uth.gr (P.M.); alexmatz@uth.gr (A.M.); 2 Hematology Laboratory, Corfu General Hospital, 49100 Corfu, Greece; mariadouska8@gmail.com; 3 Biochemical Laboratory, Venizelio Hospital, 71409 Heraklion, Greece; tsigaridakimaria@yahoo.gr; 4 Microbiological Laboratory, Kozani General Hospital” Mamatsio”, 50100 Kozani, Greece; gkiatavasiliki@gmail.com; 5 Department of Hygiene and Epidemiology, Faculty of Medicine, University of Ioannina, 45110 Ioannina, Greece; ktsilidi@uoi.gr (K.K.T.); entzani@uoi.gr (E.E.N.); 6 Center for Research Synthesis in Health, Department of Health Services, Policy and Practice, School of Public Health, Brown University, Providence, RI 02903, USA; 7 Institute of Biosciences, University Research Center of loannina, University of Ioannina, 45110 Ioannina, Greece; 8 Department of Nursing, University of Peloponnese, 22100 Tripoli, Greece; prezerpot@gmail.com; 9 Fourth Department of Internal Medicine, School of Medicine, Attikon University Hospital, National and Kapodistrian University of Athens, 12462 Athens, Greece; tsiodras@med.uoa.gr; 10 Department of Immunology and Histocompatibility, Faculty of Medicine, University of Thessaly, 41500 Larissa, Greece; maspel@med.uth.gr

**Keywords:** SARS-CoV-2, COVID-19, antibodies, IgG, seroepidemiology, long-term immune response

## Abstract

Background: Nation-wide SARS-CoV-2 seroprevalence surveys provide valuable insights into the course of the pandemic, including information often not captured by routine surveillance of reported cases. Methods: A serosurvey of IgG antibodies against SARS-CoV-2 was conducted in Greece between March and December 2020. It was designed as a cross-sectional survey repeated at monthly intervals. The leftover sampling methodology was used and a geographically stratified sampling plan was applied. Results: Of 55,947 serum samples collected, 705 (1.26%) were found positive for anti-SARS-CoV-2 antibodies, with higher seroprevalence (9.09%) observed in December 2020. Highest seropositivity levels were observed in the “0–29” and “30–49” year age groups. Seroprevalence increased with age in the “0–29” age group. Highly populated metropolitan areas were characterized with elevated seroprevalence levels (11.92% in Attica, 12.76% in Thessaloniki) compared to the rest of the country (5.90%). The infection fatality rate (IFR) was estimated at 0.451% (95% CI: 0.382–0.549%) using aggregate data until December 2020, and the ratio of actual to reported cases was 9.59 (7.88–11.33). Conclusions: The evolution of seroprevalence estimates aligned with the course of the pandemic and varied widely by region and age group. Young and middle-aged adults appeared to be drivers of the pandemic during a severe epidemic wave under strict policy measures.

## 1. Introduction

The first coronavirus disease 2019 (COVID-19) case in Greece was identified in February 2020, followed by reports of imported cases, clusters of cases, and subsequently community transmission. Greece remains affected by the COVID-19 pandemic with more than 1,000,000 cases, 20,000 deaths and a crude cumulative incidence of 9795.5 cases per 100,000 population reported through 18 December 2021 [1]. Passive case reporting remains the predominant surveillance strategy, which is prone to underestimation of disease incidence. Surveillance based on reported cases lacks the capability to accurately capture silent SARS-CoV-2 transmission from asymptomatic cases or cases with mild symptoms [2]. Furthermore, this surveillance strategy is affected by testing rates and populations’ health seeking behaviors.

Considered important for refining estimates of infection and transmission, seroprevalence surveys can be used as supplementary surveillance tools as the pandemic runs its course. Numerous seroprevalence surveys conducted throughout the world almost unanimously indicate that serum antibody derived estimations of infections are significantly higher than reported COVID-19 cases, although the seroprevalence to cumulative incidence ratios reported vary greatly across different settings [3]. The European Center for Disease prevention and Control (ECDC) pinpoints nation-wide seroprevalence studies, with a random sampling methodology, as the gold standard for monitoring population immunity. However, periodical residual blood sampling, stratified by age group and geographic area, is also proposed as a more practical approach [4].

According to the global SARS-CoV-2 seroprevalence dashboard (https://serotracker.com/ -assessed on 27 December 2021) [5], a total of 2784 seroprevalence surveys have been reported in 126 countries and territories with 25,525,121 participants. The majority of seroprevalence surveys conducted in Greece and world-wide has been limited to high-risk populations, or have not been designed for repeated sampling over time. However, nation-wide efforts are also a part of the available evidence, and testing of leftover (residual) sera coming from community clinical laboratories has offered a practical, scalable approach to estimate the prevalence of individuals who develop SARS-CoV-2 antibodies in the general population rolling over time. From March 2020 to present, an ongoing national initiative has been implemented in Greece, systematically monitoring the seroprevalence in leftover serum samples at monthly intervals, covering the vast majority of regional units in the country. Previously published reports indicate low percentages of seropositivity in Greece until August 2020 [6,7], which is in accordance with low levels of virus circulation until that period. Seroprevalence at a national scale had been consistently estimated to be lower than 0.5% for the period between March to August 2020, while the cumulative incidence of COVID-19 based on reported cases was approximately 0.1% by 30 August 2020. However, Greece experienced the first severe epidemic wave during the last quarter of 2020, beginning in September 2020 and lasting until the end of that year, when an exponential increase in reported cases, hospitalizations, and deaths was observed.

In the present study, we report the serological results of this nation-wide approach from specimens collected between March 2020 to December 2020, assessing the evolution of SARS-CoV-2 IgG antibodies seroprevalence during the pre-vaccination period (in Greece vaccination campaigns began on 27 December 2020). We also aim to understand how seroprevalence varied across different geographic regions, sexes, age groups, and periods. Moreover, we aim to estimate the true SARS-CoV-2 infections and infection fatality ratio (IFR) of the disease in Greece.

## 2. Materials and Methods

### 2.1. Study Design and Participants

The study was designed as a cross-sectional survey and repeated at monthly intervals. We used the leftover sampling methodology in order to collect serum samples (residual sera from the general population) [8]. A geographically stratified sampling plan based on regional units (NUTS level 3) was applied in order to produce a representative sample, taking into consideration both age group (0–29, 30–49, 50–69, and ≥70 years) and sex. The required sample size was determined to be 380 serum samples from each of the 13 NUTS level 2 regions, and the sample size for each regional unit (NUTS level 3) from the corresponding region was calculated according to population distribution. However, the actual number of collected samples differed from the predetermined number of samples above.

The leftover serum samples were collected from a nation-wide laboratory network, including both private microbiological laboratories as well as microbiological and bio-chemical laboratories of public hospitals, with a total of 69 laboratories participating. The samples were derived from individuals who visited the laboratories for routine screening and reasons unrelated to COVID-19.

### 2.2. Laboratory Analysis

The presence of anti-SARS-CoV-2 IgG antibodies was determined using the AB-BOTT SARS-CoV-2 IgG assay, a chemiluminescent microparticle immunoassay (CMIA), with the ARCHITECT i2000SR analyzer (Abbott, IL, USA). Anti-spike IgG antibodies are used as a marker of prior SARS-CoV-2 infection. As already stated, the method was validated in our laboratory. We used 305 pre-COVID-19 samples (obtained in 2017) as negative controls and 94 samples from patients with positive SARS-CoV-2 PCR and different symptom durations. The kit displayed 84.0% sensitivity (95% confidence interval (CI): 76.6–91.5) and 99.7% specificity (95% CI: 98.2–100). Given that vaccines were not available during the study period (May to December 2020), all positive samples for IgG anti-SARS-CoV-2 were provoked by natural infection.

### 2.3. Statistical Analysis and Calculation of Indicators

The statistical analysis applied is based on the methodology that was applied for samples between March and August 2020 [6,7], with some modifications. Analysis was performed with the use of IMB SPSS Statistics V22.0 and Microsoft Excel.

#### 2.3.1. Weighted Prevalence

Initially, we determined an unweighted relative frequency of all patient characteristics (age, sex and area of residence): this is the crude seroprevalence (S1). The weighted proportions of positive tests in the countrywide sample were based on the sex and age distribution within each regional unit (NUTS level 3) and the population of each regional unit, according to the most recent census conducted in 2011 (S2) [9]. We also adjusted the weighted proportion (S2) of positive tests to account for the accuracy (sensitivity and specificity) of the laboratory test (S3) [10,11]. Since reported COVID-19 cases were by definition outside the sampling framework, the seroprevalence was corrected taking into consideration the number of reported cases per month in accordance with the National Public Health Organization (NPHO) (S4). Therefore, we added the monthly reported cases to the estimated S3 seroprevalence in order to calculate S4. Comparison of two proportions was carried out with the ‘N-1′ chi-squared test [Campbell, I. Chi-squared and Fisher-Irwin tests of two-by-two tables with small sample recommendations. Stat. Med. 2007, 26, 3661–3675. Available online: https://pubmed.ncbi.nlm.nih.gov/17315184/ (accessed on 23 August 2021)]. For all analyses, a 5% significance level was set.

#### 2.3.2. Effective Sample Size

Since the number of collected samples from each regional unit was not proportional to the regional unit’s population, we calculated an effective sample size based on each regional unit’s population proportion, according to 2011 census data. This was done using target weighting. The methodology of the calculations is presented in Appendix A.

#### 2.3.3. Estimation of Actual Infections Based on Seroprevalence Data

In order to estimate the SARS-CoV-2 infections at specific time points, two critical assumptions were made. Specifically, it was assumed that: (i) the estimated monthly seroprevalence (%) represents the mid-day (15th) of each month and; (ii) the estimated seroprevalence reflects the actual cases at a time point 11 days earlier (4th of each month). The former assumption is based on the random distribution of the dates samples were received within a month, and the latter to evidence suggesting that “Neutralising antibodies—with the capacity to restrict virus growth in vitro—are detectable within seven to 15 days of disease onset” [12].

For the estimation of actual cases the following parameters are defined:

S*i*: Seroprevalence (%) at the month *i*

Sn*i*: Sum of new infections (as a proportion [%] of the total population) at the month *i*

Sp1*i*: Sum of previous infections (as a proportion [%] of the total population) that remain IgG positives during the month *i* of the survey

Sp2*i*: Sum of previous infections (as a proportion [%] of the total population) that are seroreversed during the month *i* of the survey

P1 (*n*): The probability to remain IgG positive *n* months after the seroconversion

P2 (*n*): The probability of seroreversion *n* months after seroconversion

I*i*: Actual cumulative cases (as a proportion [%] of the total population) at month *i*

The actual cumulative cases (as a % of the total population) at a specific month were considered equal to the seroprevalence (%) plus the sum of previous infections (%) that have been seroreserved until month *i* (Equation (1)). The sum of new infections (as a proportion [%] of the total population) at the month *i* equals the seroprevalence month *i* minus the percentage of previous infections that remained positive during the month *i* (Equation (2)):

I*i* = S*i* +Sp2*i* (1)

(2) Sni=Si−Sp1

The sum of previous infections (as a proportion [%] of the total population) that remain IgG positives during the month *i* of the survey equals the aggregate of the products of new infections (%) of each previous month multiplied by the probability of remaining positive until the month *i* (Equation (3)):

(3) $$\mathrm{Sp}1i={\mathrm{Sn}}_{i-1}*P1_{1}+{\mathrm{Sn}}_{i-2}*P1_{2}+\cdots ,\;Sn_{1}*P1_{i-1}$$

The sum of previous infections (as a proportion [%] of the total population) that are seroreversed during the month *i* is equal to the aggregate of the products of new infections (%) of each previous month multiplied by the probability of seroreversion until the month *i* (Equation (4)):

(4) $$\mathrm{Sp}2i={\mathrm{Sn}}_{i-1}*P2_{1}+{\mathrm{Sn}}_{i-2}*P2_{2}+\cdots ,\;Sn_{1}*P2_{i-1}$$

The probabilities P1 and P2 were estimated assuming that 15.26% of seropositives seroreverse within a month. This assumption is based on the results of a study of seropositive health care personnel recruited from 13 hospitals, which found that 28.2% experienced seroreversion within 60 days [13]. The presumed seroreversion pattern is presented in Appendix B.

By solving the Equations (1)–(4), we calculated the cumulative incidence at eight distinct time points (April to December 2020).

#### 2.3.4. Calculation of Case Fatality Ratio and Infection Fatality Ratio

The case fatality ratio (CFR) for each month was calculated by dividing the number of deaths attributed to COVID-19 and reported to the NPHO, by the number of cases reported to the NPHO. The 95% CI for weighted data were estimated using normal approximation of binomial distribution and effective sample size, rather than the collected sample size (further explained below). It should be noted that clusters of cases from refugee camps and from a cruise ferry—which were not considered community cases (302 cases in total)—were excluded from analysis for CFR.

The infection fatality ratio (IFR) is the ratio of deaths to the number of estimated individuals infected with SARS-CoV-2. The IFR and corresponding 95% CI were calculated using actual infections (and the corresponding 95% CI) derived from the S1, S2, S3, and S4 seroprevalence, with the methodology described in Section 2.3.3.

## 3. Results

During the study period from March to December 2020, a total of 55,947 residual, random leftover serum samples were collected and analyzed. Of the total number of samples, 32,207 (57.57%) were derived from females. Regarding the age distribution of the study population, 10,850 (19.39%) belonged to the “0–29” age group, 15,686 (28.04%) to the “30–49” group, 14,682 (26.24%) to the “50–69” group and 11,441 (20.45%) to the “70+” group. In total, 705 samples were found positive for anti-SARS-CoV-2 IgG antibodies. We calculated the crude seroprevalence (S1), the age, sex and population-adjusted seroprevalence (S2), the sensitivity and specificity-adjusted seroprevalence (S3), and the modified for reported cases seroprevalence (S4) for each month by sex and age group. The detailed results for September, October, November, and December 2020 are presented in Supplementary Table S1, Table S2, Table S3 and Table S4 (respectively). The geographic distribution of leftover samples is presented in Supplementary Figure S1.

**Figure 1:**
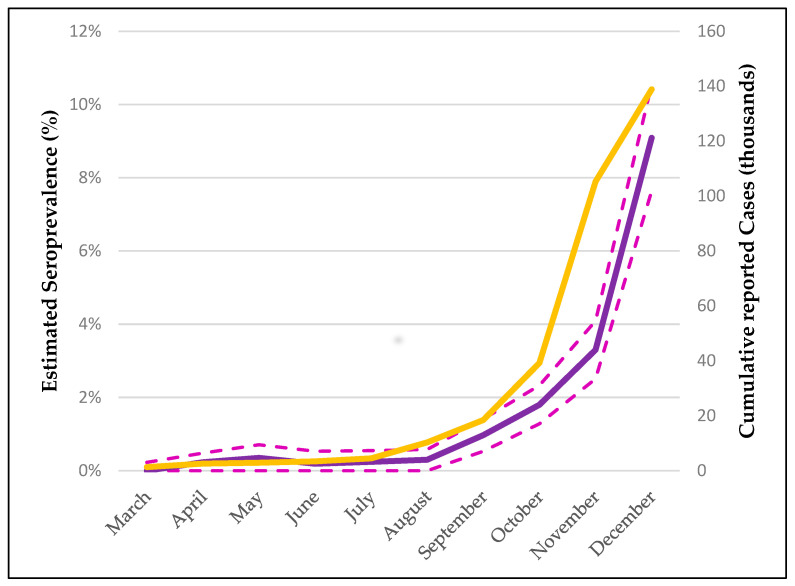
Evolution of estimated monthly seroprevalence (S3) from March to December 2020, in comparison with reported COVID-19 cumulative cases. Purple line represents the estimated seroprevalence (%). Dashed purple lines represent the upper and lower limits of the 95% Confidence intervals. Yellow line represents the cumulative cases as a percentage of the total population.

Figure 1 presents the monthly S3 seroprevalence (%, 95% CI) in parallel with the cumulative COVID-19 cases reported for each month, from the beginning of the pandemic until December 2020. A significant increase in seroprevalence is observed beginning in September 2020, followed by a sharp (approximately 3-fold) increase in December 2020 when the highest seropositivity of 9.09% was calculated (95% CI: 7.58–11.41%). Similarly, cumulative reported cases increased rapidly; however, the highest increase is observed earlier (November 2020).

Based on the monthly seroprevalence, it was estimated that 10.04% of the Greek population was infected with SARS-CoV-2 since the beginning of the pandemic until 4 December 2020. Of those estimated as infected, only 1.05% have been diagnosed with COVID-19 and reported to the NPHO, corresponding to a ratio of infections to reported cases of 9.59 (95% CI: 7.88–11.33). Table 1 presents for each month the estimated proportions of seropositives, newly infected, previously infected that remain seropositive, previously infected that are seroreversed, and total (cumulative) infected. All mentioned proportions are expressed as percentages (%) of the total population with the corresponding 95% CIs. Based on the assumptions formulated in Section 2.3.3, the above parameters correspond to the 4th of each month. The estimated ratio of infections to reported cases was characterized by a high variance during the earlier months, where SARS-CoV-2 circulation in Greece was low. This low incidence caused considerable uncertainty in the estimations, with 95% CIs often including zero. However, the increase of SARS-CoV-2 incidence from September 2020 and onwards lead to more precise estimations. A decreasing trend in the infected/reported ratio is observed during the study period (Supplementary Figure S2). The CFR also increased from 2.12% (95% CI: 1.91–2.32%) in September to 3.48% (95% CI: 3.39–3.58%) in December. Concerning the IFR, the overall estimation was 0.451% (0.382–0.549%) based on S3 prevalence and using cumulative data from December 2020. In Supplementary Table S5, the evolution of the IFR in Greece is presented by using the S1, S2, S3 and S4 prevalence.

**Figure 2:**
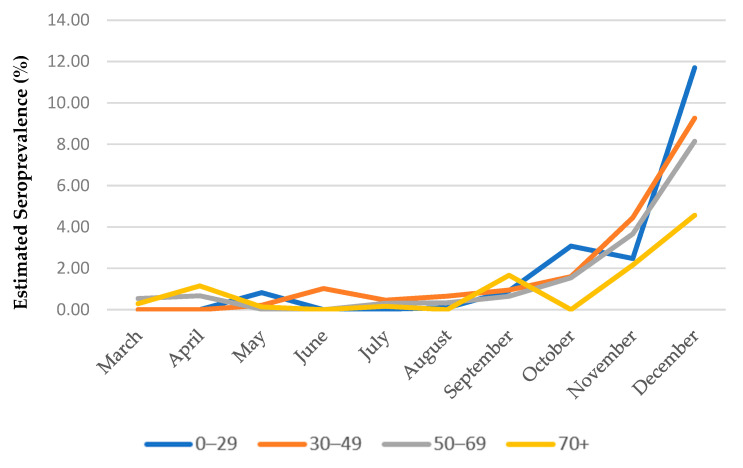
Evolution of estimated monthly seroprevalence (S3) by age group from March to December 2020.

The comparison of seropositivity across population groups with different demographic characteristics revealed significant variance between age groups and geographical areas. While in May 2020 females presented higher seroprevalence compared to males, this difference was reversed in August 2020 when seroprevalence was higher in males. This the association remained significant in October and November 2020 (Supplementary Tables S2 and S3); however, no significant difference is reported for December 2020 where seroprevalence was estimated at 8.67% in females, compared to 9.49% in males (*p* = 0.226). Older individuals (“70+” age group) had consistently lower levels of seropositivity from May 2020 and onwards compared to other age groups. Figure 2 presents the seroprevalence by age group over time. It is considered noteworthy that the “0–29” age group showed the most rapid (almost 5-fold) increase, from 2.47% in November to 11.70% in December, which was the highest among all age groups during that month.

**Figure 3:**
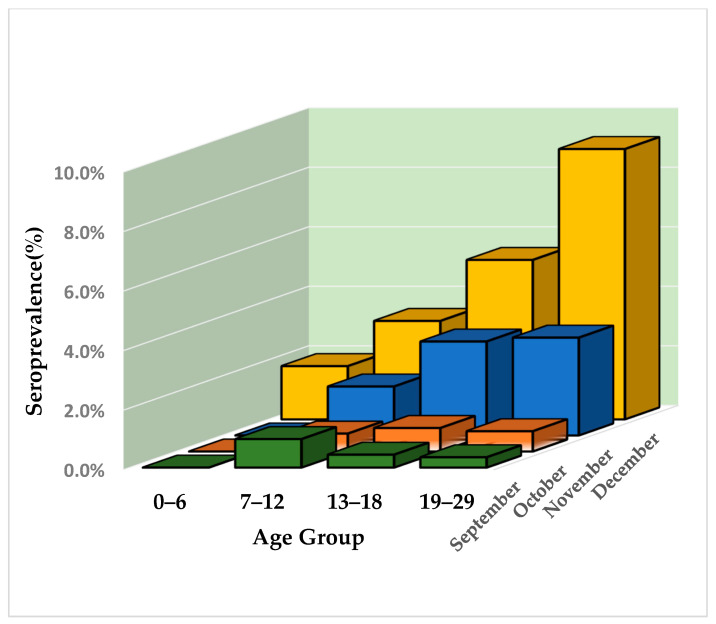
Estimated monthly seroprevalence (S1, adjusted for sensitivity and specificity) by age group in individuals <29 years of age from September to December 2020.

To explore the above observation, we present the seroprevalence of the “0–29” age group further stratified by 4 age groups, specifically: 0–6 years, 7–12 years, 13–18 years and 19–29 years (Figure 3). It is evident that as the pandemic evolved, young adults (19–29 years of age) were more frequently identified as positive for the presence of SARS-CoV-2 antibodies. A clear pattern is established in November 2020 and particularly in December 2020, where seroprevalence increases with age.

Table 2 presents the seroprevalence estimations of 5 major geographical areas representing the entire country. In December 2020, Attica had the highest prevalence (11.92%), followed by northern Greece (10.34%) and central Greece (5.81%). Southern Greece and the islands had significantly lower seroprevalence (2.53% and 1.74%, respectively). It should also be noted that these specific geographical areas (islands, southern Greece) do not conform to the national pattern observed of a rapid increase in seroprevalence in December 2020; seropositivity in these areas increased in November but remained relatively stable in December. On the contrary, in Attica, northern Greece, and central Greece, the substantial increase in December is clear. Since a large proportion of the Greek population resides in the highly populated metropolitan areas of Athens and Thessaloniki, we also compared seropositivity in these areas to the rest of the country (Table 3). It was observed that in the Regional Units of Attica (which include the city of Athens) and Thessaloniki (which include the city of Thessaloniki), seroprevalence was approximately two times higher compared to the rest of the country in December 2020. It is also observed that in the Region of Thessaloniki, seropositivity was lower in September and December 2020 compared to other regions, indicating a more rapid spread in this specific area.

## 4. Discussion

In a nation-wide repeated SARS-CoV-2 seroprevalence survey, we tested 55,947 leftover specimens submitted for non–SARS-CoV-2 testing from March to December 2020, covering the vast majority (67 out of 74) of Greece’s Regional Units. For the majority of prefectures, fewer than 10% of the Greek population had evidence of previous SARS-CoV-2 infection using currently available commercial IgG assays. The estimated seroprevalence of 9.09% as of December 2020 is close to the global average for that period, which was estimated at 9.47% (95% CI 8.99–9.95%) in a meta-analysis by Rostami et al. [14]. Seroprevalence in Greece evolved over time and varied across geographic regions and between metropolitan/non-metropolitan areas, with regional units encompassing the cities of Athens and Thessaloniki showing an approximate two-fold increase in seropositivity. Our present work extends from our previously published seroprevalence survey reports, and is in accordance with the limited number of published studies from Greece in high-risk populations, specifically considering health care workers, transmission hot spots or other unique groups such as blood donors [15,16,17,18,19,20,21]. These studies all addressed earlier stages of the COVID-19 pandemic. By testing for SARS-CoV-2 antibodies across the whole age spectrum and within an outpatient clinical care setting, we were better positioned to assess seroprevalence in the general population. Moreover, our study includes a considerable proportion of samples tested from individuals living in non-metropolitan areas, which follows the distribution of the Greek population and achieves a wider geographical representation. Regional estimates are also valuable as they correspond to different background estimates of risk assessment and different containment strategies. Our work contributes to a growing body of evidence across the globe examining population-level SARS-CoV-2 exposure in the pre-vaccination pandemic period, as well as transmission variation across age groups and geographical areas. Corroborating other large-scale seroprevalence surveys conducted in countries hit harder by the pandemic, such as the USA [22], the United Kingdom [23], and Spain [24], we report that most individuals in Greece did not have evidence of previous SARS-CoV-2 infection, even following the third pandemic wave.

As observed in other surveys [22,25], we found SARS-CoV-2 seroprevalence across nearly all prefectures to be lower in older adults (age group: 70+) compared with younger adults a finding that became more clear from October and onwards. It is noteworthy that this finding cannot be derived from routine surveillance data based on reported cases, where cumulative incidence by the end of 2020 in the age group “65+” was close to the overall cumulative incidence across all ages (1.21% vs. 1.28%). This indicates that middle-aged and younger individuals were more likely to escape diagnosis and reporting, when compared to older individuals. According to a systematic review published by Huang et al. in late 2020 [26] “for endemic coronaviruses, seroprevalence rises sharply in childhood, with little to no change in seroprevalence by age among adults”. The World Health Organization (WHO) “Scientific brief on the COVID-19 disease in children and adolescents” states that children less than 10 years of age are less frequently infected when compared to older children and adults, but seroprevalence in adolescents is similar to adults [27]. Seroprevalence among children, adolescents, and young adults in our survey demonstrated a clear pattern of increasing positivity with age in December 2020, when the overall seropositivity was highest. This may be explained by the strict restrictions applied in November, which included the closure of schools. In general, our results indicate that young adults and middle-aged individuals were the main drivers of the pandemic during the study period, since both the elderly and children were less frequently detected as positive for SARS-CoV-2 antibodies. It is also interesting that in October and November 2020, gender was strongly correlated to seropositivity, with males being infected more frequently than females. However, analysis of data from December 2020—which presumably reflected the situation during an upsurge of COVID-19 infections—did not reveal any significant difference in seroprevalence between genders.

The interpretation of our results indicates that throughout the pandemic, the distribution of SARS-CoV-2 infections across population characteristics as well as the epidemiological attributes of the disease are continuously changing. Our study therefore highlights the importance of conducting repeated sero-surveillance, as a strategy to monitor and study the way infection patterns evolve over time.

An additional application of repeated seroprevalence studies is the ability to calculate the ratio of infections to reported SARS-CoV-2 cases. We estimated this ratio as 9.59, which is comparable to reports in the literature [25], although direct comparisons are considered risky. A recently published meta-analysis by Bobrovitz et al. (2021) estimated the ratio between seroprevalence estimates and the corresponding cumulative incidence of SARS-CoV-2 infection nine days prior at 18.1 (IQR 5.9–38.7) by synthesizing data from 49 national-wide studies [25]. This ratio seems to vary greatly in both spatial and temporal terms, and may be affected by multiple parameters including differences in the health care systems’ structure and in the implementation levels of national strategies. However, monitoring relative changes over time—both nationally and within a region—can provide a complementary measure to assessing testing capacity and other metrics of public health policy. Our results demonstrate a gradual decline in the infected/reported cases ratio, which was higher during the first months of the pandemic. The most likely explanation for this observed trend is the substantial increase in the testing rate that occurred at the end of 2020 compared to the first months of the pandemic.

Although seroprevalence surveys often indicate a higher burden of infection than reported cases alone, they may still underestimate, repeated notwithstanding, the total number of prior infections. Immune response may vary at the individual level besides whether an asymptomatic or mild infection occurred and declines in SARS-CoV-2 antibodies following infection have been observed. In the present study, we estimated the actual infections, and consequently the IFR, considering the probability of seroreversion of previously infected individuals. We assumed a seroreversion pattern based on a study conducted in 2020 that examined the decline in SARS-CoV-2 antibodies following mild infection. Relying on a single study to derive a seroreversion pattern is a limitation of the present work. The kinetics of waning antibodies appear to differ by type of assay, viral target and severity of infection.

Although the present periodical sero-surveillance study has significant strengths, it is not without limitations while the overall direction of bias resulting from these limitations is unclear. Individuals who undergo blood testing for routine screening or clinical care may not accurately represent the general Greek population. They can differ with regard to their underlying health status, exposure risk, COVID-19 risk, risk perception and awareness, health-related behaviors, access to care, or adherence to prevention measures such as use of face masks, hand hygiene, and physical distancing. The geographic catchment of samples was dependent on the location of the testing laboratories, which predominantly draw their population form urban areas. Moreover, despite the large study size, we were not able to reach our target sample numbers in all age groups or regions. Finally, specimens were tested using a single immunoassay with distinct performance characteristics, although appropriate adjustments have been made for assay performance.

## 5. Conclusions

In this nation-wide sero-surveillance study in Greece, we found that seroprevalence estimates varied widely across different periods, geographical areas, and age groups. The rapid increase in seropositivity at the end of 2020 resembled the increasing pattern of reported cases. Young and middle-aged adults appeared to be drivers of the pandemic during a severe epidemic wave under strict policy measures. The ratio of infected to reported cases declined over time, although it remained high as of December 2020. Our results reinforce the need for continued systematic sero-surveillance rather than single surveys at specific time points, since the epidemiological attributes of COVID-19 are continuously evolving.

## Figures and Tables

**Table 1:** Estimated proportion (%, 95% CI) of (actual) new, previous, and total cumulative infections for each month based on seroprevalence (S3) data.

Month	Seropositivity	New Infections	Previous Infections (IgG Positive)	Previous Infections (Seroreversed)	Total Infections	Cumulative Cases *	Ratio (Total/Reported)
September	0.97 (0.53–1.41)	0.72 (0.53–0.92)	0.57 (0.10–1.18)	0.20 (0.04–0.40)	1.17 (0.57–1.81)	0.104	11.25 (5.43–17.37)
October	1.80 (1.28–2.33)	0.98 (0.83–1.14)	0.82 (0.45–1.19)	0.35 (0.12–0.61)	2.15 (1.40–2.94)	0.183	11.74 (7.63–16.08)
November	3.30 (2.51–4.09)	1.77 (1.43–2.12)	1.53 (1.08–1.97)	0.51 (0.22–0.83)	3.81 (2.73–4.92)	0.434	8.77 (6.28–11.33)
December	9.09 (7.64–10.55)	6.29 (5.51–7.08)	2.80 (2.13–3.47)	0.95 (0.61–1.32)	10.04 (8.25–11.87)	1.047	9.59 (7.88–11.33)

All parameters are presented as proportions (%) of the total population * Cumulative cases correspond to the 4th of each month.

**Table 2:** Monthly seropositivity (S3) in five major geographical units.

	Central Greece (Thessaly & Central Greece)			Islands			Northern Greece (Macedonia & Thrace & Epirus)			Southern Greece (Western Greece & Peloponnese)			Attica		
Month	p (S3)	95% CI		p (S3)	95% CI		p (S3)	95% CI		p (S3)	95% CI		p (S3)	95% CI	
September	0.31%	0.00%	0.97%	0.56%	0.00%	1.29%	0.77%	0.27%	1.28%	0.06%	0.00%	0.32%	1.54%	0.72%	2.35%
October	0.44%	0.00%	0.90%	0.47%	0.00%	1.22%	0.78%	0.30%	1.25%	0.00%	0.00%	0.21%	2.29%	1.35%	3.23%
November	1.30%	0.67%	1.94%	1.69%	0.58%	2.80%	3.68%	2.76%	4.60%	2.76%	1.76%	3.77%	3.40%	1.70%	5.09%
December	5.81%	4.57%	7.05%	1.74%	0.85%	2.63%	10.34%	8.32%	12.37%	2.53%	1.42%	3.64%	11.92%	8.69%	15.15%

**Table 3:** Monthly seropositivity (S3) in the Regional Units of Attica and Thessaloniki compared to the rest of the country.

	Rest of Country					Attica					Thessaloniki				
	Pos	Total	S3 (%)	95% CI		Pos	Total	S3 (%)	95% CI		Pos	Total	S3 (%)	95% CI	
September	39	6283	0.65	0.16	1.14	19	1288	1.54	0.72	2.35	1	218	0.19	0.00	1.26
October	46	6520	1.80	1.12	2.47	31	1413	2.29	1.37	3.21	1	227	0.12	0.00	1.10
November	123	5797	3.08	2.27	3.88	20	620	3.40	1.76	5.04	21	652	3.76	2.09	5.44
December	247	5503	5.90	4.56	7.24	60	603	11.92	9.03	14.82	14	125	12.76	6.21	19.30

## Data Availability

The data are not publicly available as they contain sensitive information at individual level.

## Supplementary Materials

The following are available online at https://www.mdpi.com/article/10.3390/diagnostics12020295/s1, Figure S1: Geographical distribution of leftover samples collected for COVID-19 serosurvey. Figure S2: Ratio of Infected individuals to reported cases during the study period. Table S1: Anti-SARS-CoV-2 IgG antibody seroprevalence, Greece, September 2020, Table S2: Anti-SARS-CoV-2 IgG antibody seroprevalence, Greece, October 2020, Table S3: Anti-SARS-CoV-2 IgG antibody seroprevalence, Greece, November 2020, TableS4: Anti-SARS-CoV-2 IgG antibody seroprevalence, Greece, December 2020, Table S5: Infection Fatality rates (deaths per 1000 infections) for each month calculated by using different estimations of seroprevelnce (S1, S2, S3, S4).

## Appendix A

The target sample size for a regional unit *i* is t*_i_*, and the actual sample size for the regional unit *i* is a***_i_***. The weighting factor for the regional unit *i* is calculated with the following formula:

(A1) $$f_{i}=\frac{t_{i}}{a_{i}}$$

The weighted sample size (w*_i_*) for the regional unit *i* is calculated as follows:

w*_i_* = t*_i_* × f*_i_* (A2)

For k regional units and a countrywide target sample size of n*_t_*, the country-wide effective sample size (n*_e_*) is calculated with the following formula:

(A3) $$n_{e}=\frac{n_{t}{}^{2}}{\sum_{i=1}^{k}w_{i}}$$

This can also be written as:

(A4) $$n_{e}=\frac{{\left(\sum_{i=1}^{k}t_{i}\right)}^{2}}{\sum_{i=1}^{k}\frac{t_{i}{}^{2}}{a_{i}}}$$

## Appendix B

**Table A1:** Assumed probabilities of remaining IgG positive (P1) and seroreverting (P2) *n* months after seroconverstion.

Probability of Remaining IgG Positive (Months Since Seroconvertion)		Probability of Seroreversion (Months Since Seroconvertion)	
P1(0)	100.0%	P2(0)	0.0%
P1(1)	84.7%	P2(1)	15.3%
P1(2)	71.8%	P2(2)	28.2%
P1(3)	60.9%	P2(3)	39.1%
P1(4)	51.6%	P2(4)	48.4%
P1(5)	43.7%	P2(5)	56.3%
P1(6)	37.0%	P2(6)	63.0%
P1(7)	31.4%	P2(7)	68.6%
P1(8)	26.6%	P2(8)	73.4%
