# Supplementary material for: SARS-CoV-2 Sero-Surveillance in Greece: Evolution over Time and Epidemiological Attributes during the Pre-Vaccination Pandemic Era
Source: Diagnostics (Basel). 2022 Jan 25;12(2):295. doi: 10.3390/diagnostics12020295 (PMC8871128; doi:10.3390/diagnostics12020295)
Supplement: Supplementary file 1 [file diagnostics-12-00295-s001.zip › diagnostics-1563427-supplementary/Figure SF2 Ratio of Infected individuals to reported cases during the study period.pdf]

Figure SF2: Ratio of Infected individuals to reported cases during the study period.

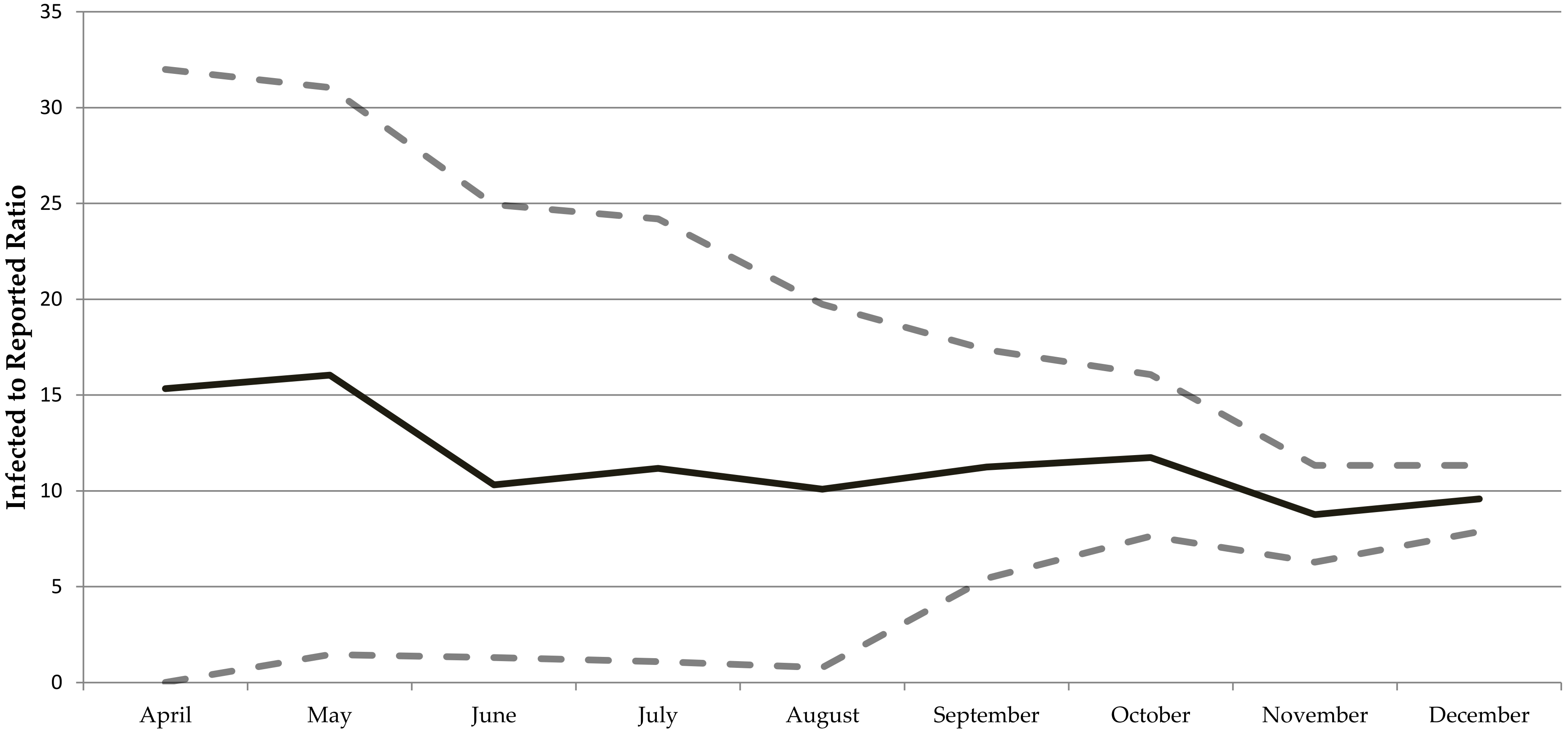

Dashed lines represent the high and low 95% Confidence intervals
