# Supplementary material for: SARS-CoV-2 Sero-Surveillance in Greece: Evolution over Time and Epidemiological Attributes during the Pre-Vaccination Pandemic Era
Source: Diagnostics (Basel). 2022 Jan 25;12(2):295. doi: 10.3390/diagnostics12020295 (PMC8871128; doi:10.3390/diagnostics12020295)
Supplement: Supplementary file 1 [file diagnostics-12-00295-s001.zip › diagnostics-1563427-supplementary/supplementary tables.pdf]

Table ST1. Anti-SARS-CoV-2 IgG antibody seroprevalence, Greece, September 2020

**September**		**Positive/ sample size**	**S1: Crude prevalence**		**S2: Age, sex and population-adjusted prevalence**		**S3: S2 + adjustment for sensitivity and specificity**		**S4: S3 + NPHO data**	
		n/N	Prevalence (%)	95% CI	Prevalence (%)	95% CI	Prevalence (%)	95% CI	Prevalence (%)	95% CI
**Total**		59/7789	0,76	0.56–0.95	1,11	0.75-1.48	0,97	0.53-1.41	1,15	0.71-1.59
**Age group (years)**	0–29	13/1935	0,67	0.31-1.04	1,06	0.38-1.75	0,91	0.09-1.73	1,06	0.24-1.88
	30–49	20/2186	0,91	0.52 -1.31	1,1	0.42-1.77	0,95	0.14-1.76	1,16	0.35-1.97
	50–69	12/2121	0,57	0.25-0.88	0,84	0.21-1.47	0,65	0.00-1.40	0,83	0.18-1.58
	≥ 70	14/1547	0,9	0.43-1.38	1,69	0.48-2.90	1,67	0.22-3.11	1,78	0.33-3.22
**Sex**	Male	25/3043	0,82	0.50-1.14	1,05	0.49-1.61	0,89	0.22-1.57	1,09	0.41-1.76
	Female	35/4746	0,72	0.48-0.96	1,17	0.68-1.67	1,04	0.46-1.63	1,19	0.60-1.78
**'N-1' chi-squared test Difference between sex**			Difference = 0.10% p = 0.620		Difference = 0.12% p = 0.624		Difference = 0.15% p = 0.512		Difference = 0.10% p = 0.686	

Table ST2. Anti-SARS-CoV-2 IgG antibody seroprevalence, Greece, October 2020

**October**		**Positive/ sample size**	**S1: Crude prevalence**		**S2: Age, sex and population-adjusted prevalence**		**S3: S2 + adjustment for sensitivity and specificity**		**S4: S3 + NPHO data**	
		n/N	Prevalence (%)	95% CI	Prevalence (%)	95% CI	Prevalence (%)	95% CI	Prevalence (%)	
**Total**		78/8160	0.96	0.74-1.17	1.81	1.37-2.25	1.80	1.28–2.33	2.16	
**Age group (years)**	0–29	13/1630	0.80	0.37-1.23	2.87	1.76-3.98	3.07	1.74-4.40	3.40	
	30–49	30/2342	1.28	0.83-1.74	1.64	0.87-2.41	1.60	0.68-2.52	2.04	
	50–69	25/2292	1.09	0.67-1.52	1.59	0.76-2.41	1.54	0.56-2.52	1.91	
	≥ 70	10/1896	0.53	0.20-0.85	0.19	0-0.56	0.00	0.00-0.31	0.08	
**Sex**	Male	41/3401	1.21	0.84-1.57	2.92	2.08-3.75	3.12	2.13-4.12	3.51	
	Female	37/4759	0.78	0.53-1.03	0.75	0.38-1.13	0.54	0.09-1.00	0.86	
**'N-1' chi-squared test Difference between sex**			Difference = 0.43% p = 0.05		Difference = 2.17% p <0.001		Difference = 2.58% p <0.001		Difference = 2.65% p <0.001	

Table ST3. Anti-SARS-CoV-2 IgG antibody seroprevalence, Greece, November 2020

**November**		**Positive/ sample size**	**S1: Crude prevalence**		**S2: Age, sex and population-adjusted prevalence**		**S3: S2 + adjustment for sensitivity and specificity**		**S4: S3 + NPHO data**	
		n/N	Prevalence (%)	95% CI	Prevalence (%)	95% CI	Prevalence (%)	95% CI	Prevalence (%)	95% CI
**Total**		164/7069	2.32	1.97–2.67	3.06	2.40-3.72	3.30	2.51-4.09	4.28	3.49-5.07
**Age group (years)**	0–29	40/1456	2.75	1.91-3.59	2.37	1.06-3.68	2.47	0.90-4.04	3.25	1.68-4.82
	30–49	58/2108	2.75	2.05 -3.45	4.03	2.59-5.47	4.45	2.73-6.17	5.61	3.89-7.33
	50–69	44/2057	2.14	1.51-2.76	3.37	2.17-4.57	3.67	2.23-5.10	4.82	3.38-6.26
	≥ 70	22/1448	1.52	0.89-2.15	2.11	0.73-3.48	2.16	0.52-3.80	2.95	1.31-4.59
**Sex**	Male	78/2975	2.62	2.05-3.20	3.62	2.50-4.73	3.96	2.63-5.30	5.02	3.69-6.35
	Female	86/4094	2.10	1.66-2.54	2.52	1.73-3.31	2.65	1.70-3.60	3.59	2.64-4.54
**'N-1' chi-squared test Difference between sex**			Difference = 0.52% p = 0.152		Difference = 1.10% p =0.007		Difference = 1.31% p =0.002		Difference = 1.43% p =0.003	

Table ST4. Anti-SARS-CoV-2 IgG antibody seroprevalence, Greece, December 2020

December		Positive/ sample size	S1: Crude prevalence		S2: Age, sex and population-adjusted prevalence		S3: S2 + adjustment for sensitivity and specificity		S4: S3 + NPHO data	
		n/N	Prevalence (%)	95% CI	Prevalence (%)	95% CI	Prevalence (%)	95% CI	Prevalence (%)	95% CI
**Total**		321/6231	5.15	4.60-5.70	7.91	6.70-9.13	9.09	7.64-10.55	10.40	8.95-11.85
**Age group (years)**	0–29	79/1336	5.91	4.65-7.18	10.09	7.46-12.73	11.70	8.56-14.85	12.67	9.52-15.81
	30–49	108/1847	5.85	4.78 -6.92	8.05	5.76-10.34	9.26	6.52-12.00	10.77	8.03-13.50
	50–69	98/1729	5.67	4.58-6.76	7.11	4.93-9.30	8.14	5.54-10.75	9.72	7.11-12.32
	≥ 70	36/1319	2.73	1.85-3.61	4.13	1.74-6.51	4.57	1.72-7.42	5.75	2.90-8.61
**Sex**	Male	130/2671	4.87	4.05-5.68	7.56	5.65-9.46	8.67	6.39-10.94	10.05	7.78-12.33
	Female	191/3560	5.37	4.62-6.11	8.24	6.64-9.85	9.49	7.58-11.41	10.76	8.85-12.68
**'N-1' chi-squared test Difference between sex**			Difference = 0.50% p = 0.377		Difference = 0.68% p =0.326		Difference = 0.82% p =0.226		Difference = 0.71% p =0.365	

Table ST5: Infection Fatality rates(deaths per 1,000 infections) for each month calculated by using different estimations of seroprevelnce (S1,S2,S3,S4)

	s1			s2			S3			S4		
	IFR	LOWER LIMIT	UPPER LIMIT	IFR	LOWER LIMIT	UPPER LIMIT	IFR	LOWER LIMIT	UPPER LIMIT	IFR	LOWER LIMIT	UPPER LIMIT
**APRIL**	3,10	2,13	5,65	2,65	1,86	4,48	5,65	2,71	-	5,20	2,60	65,00
**MAY**	2,75	2,32	4,60	2,11	1,65	4,47	4,15	2,15	45,59	3,68	2,03	20,46
**JUNE**	2,78	2,23	4,71	2,22	1,62	5,37	6,46	2,67	51,28	4,67	2,14	25,61
**JULY**	2,18	1,74	3,56	1,94	1,41	4,22	5,31	2,45	54,13	3,85	1,86	24,88
**AUGUST**	2,39	1,73	4,57	2,13	1,50	4,71	5,51	2,81	71,23	3,75	1,88	20,94
**SEPTEMBER**	2,57	2,03	3,94	1,96	1,46	4,34	3,16	2,05	6,55	2,36	1,53	4,66
**OCTOBER**	3,16	2,54	4,56	2,01	1,57	3,14	2,75	2,01	4,23	2,00	1,37	3,02
**NOVEMBER**	6,54	5,53	8,33	5,07	4,03	7,32	5,91	4,58	8,25	4,20	3,32	5,60
**DECEMBER**	6,38	5,70	7,39	4,45	3,80	5,55	4,51	3,82	5,49	3,67	3,13	4,40
